# Effective photo-enhancement of cellular activity of fluorophore-octaarginine antisense PNA conjugates correlates with singlet oxygen formation, endosomal escape and chromophore lipophilicity

**DOI:** 10.1038/s41598-017-18947-x

**Published:** 2018-01-12

**Authors:** Reza Yarani, Takehiko Shiraishi, Peter E. Nielsen

**Affiliations:** Department of Cellular and Molecular Medicine, Faculty of Health and Medical Sciences, University of Copenhagen, Copenhagen, Denmark

## Abstract

Photochemical internalization (PCI) is a cellular drug delivery method based on the generation of light-induced reactive oxygen species (ROS) causing damage to the endosomal membrane and thereby resulting in drug release to the cytoplasm. In our study a series of antisense fluorophore octaarginine peptide nucleic acid (PNA) conjugates were investigated in terms of PCI assisted cellular activity. It is found that tetramethylrhodamine and Alexa Fluor 555 conjugated octaarginine PNA upon irradiation exhibit more than ten-fold increase in antisense activity in the HeLa pLuc705 luciferase splice correction assay. An analogous fluorescein conjugate did not show any significant enhancement due to photobleaching, and neither did an Alexa Fluor 488 conjugate. Using fluorescence microscopy a correlation between endosomal escape and antisense activity was demonstrated, and in parallel a correlation to localized formation of ROS assigned primarily to singlet oxygen was also observed. The results show that tetramethylrhodamine (and to lesser extent Alexa Fluor 555) conjugated octaarginine PNAs are as effectively delivered to the cytosol compartment by PCI as by chloroquine assisted delivery and also indicate that efficient photodynamic endosomal escape is strongly dependent on the quantum yield for photochemical singlet oxygen formation, photostability as well as the lipophilicity of the chromophore.

## Introduction

Cell penetrating peptides (CPPs) are being developed as effective carriers for intracellular delivery of large hydrophilic molecules, such as polypeptides, proteins and nucleic acids. However, most CPPs exploit endocytotic pathways as the main uptake route, and their efficacy is therefore generally limited due to endosomal entrapment and poor endosomal escape. Improved endosomal release can be achieved by auxiliary agents such as chloroquine (CQ) or Ca^2+^ or by photochemical internalization (PCI) using photosensitizers via their photo-induced excited triplet state generated singlet oxygen^1–5^.

Compared to other methods for enhancing endosomal escape (cytosolic delivery), PCI has the advantage that the effect of the drug may be spatially and temporally controlled even *in vivo* via targeted radiation^6–10^. Photosensitizers exploited in PCI are usually hydrophobic/amphiphilic compounds that localize preferably in cell - including endosomal and lysosomal - membranes^11^. This approach can increase controlled endosomal escape of entrapped compounds upon irradiation with light of appropriate wavelength. The endosomal escape is caused by photosensitizer-induced ROS (primarily singlet oxygen (^1^O_2_))^12–15^ that oxidize lipids in the membrane, thereby destabilizing the lipid bilayer and consequently causing increased leakage through the membrane. Singlet oxygen has an extremely short half-life (∼0.01–0.04 μs) and therefore the effect is very local^16^. Numerous studies have highlighted the promise of using PCI for intracellular delivery of both nucleic acids and proteins^11,17,18^, and the technique is parallel to photodynamic therapy, which is already used in the clinic^19,20^.

The structural DNA-mimic, peptide nucleic acid (PNA) is of interest in its own right as a third generation antisense agent, but has also been widely used as a model for studying cellular (and to a lesser extent *in vivo*) delivery modalities^3,4^. It has previously been shown that cellular delivery of CPP-PNA conjugates can be enhanced by using PCI, through an auxiliary photosensitizer^5,21–23^ or a covalently conjugated dye^24,25^. In the present work, we extend these studies by using fluorophore (Alexa Fluor 488 (AF488), Fluorescein, Alexa Fluor 555 (AF555) and Tetramethylrhodamine (TMR)) octaarginine PNA conjugates. This approach allowed us to follow the cellular localization of the conjugate, the biological antisense effect as well as the production of singlet oxygen in parallel. Our results show that the antisense activity of TMR and AF555 conjugated octaarginine PNA in the HeLa pLuc705 luciferase splice correction assay is increased more than ten-fold upon irradiation, while an analogous (photolabile) fluorescein conjugate did not yield any significant enhancement due to photobleaching, and neither did a photostable AF488 conjugate.

## Results and Discussion

A series of four fluorophore CPP-PNA conjugates were designed based on a well characterized 18-mer antisense PNA that targets the cryptic splice site of the engineered luciferase pre-mRNA of the extensively used HeLa pLuc705 cell antisense assay system^26,27^. The PNA was conjugated to the well-studied CPP octaarginine^28–30^, and we chose the widely used fluorescein and TMR fluorophores as well as the more photostable Alexa Fluor homologues AF488 and AF555.

### PNA antisense activity

**Figure 1:**
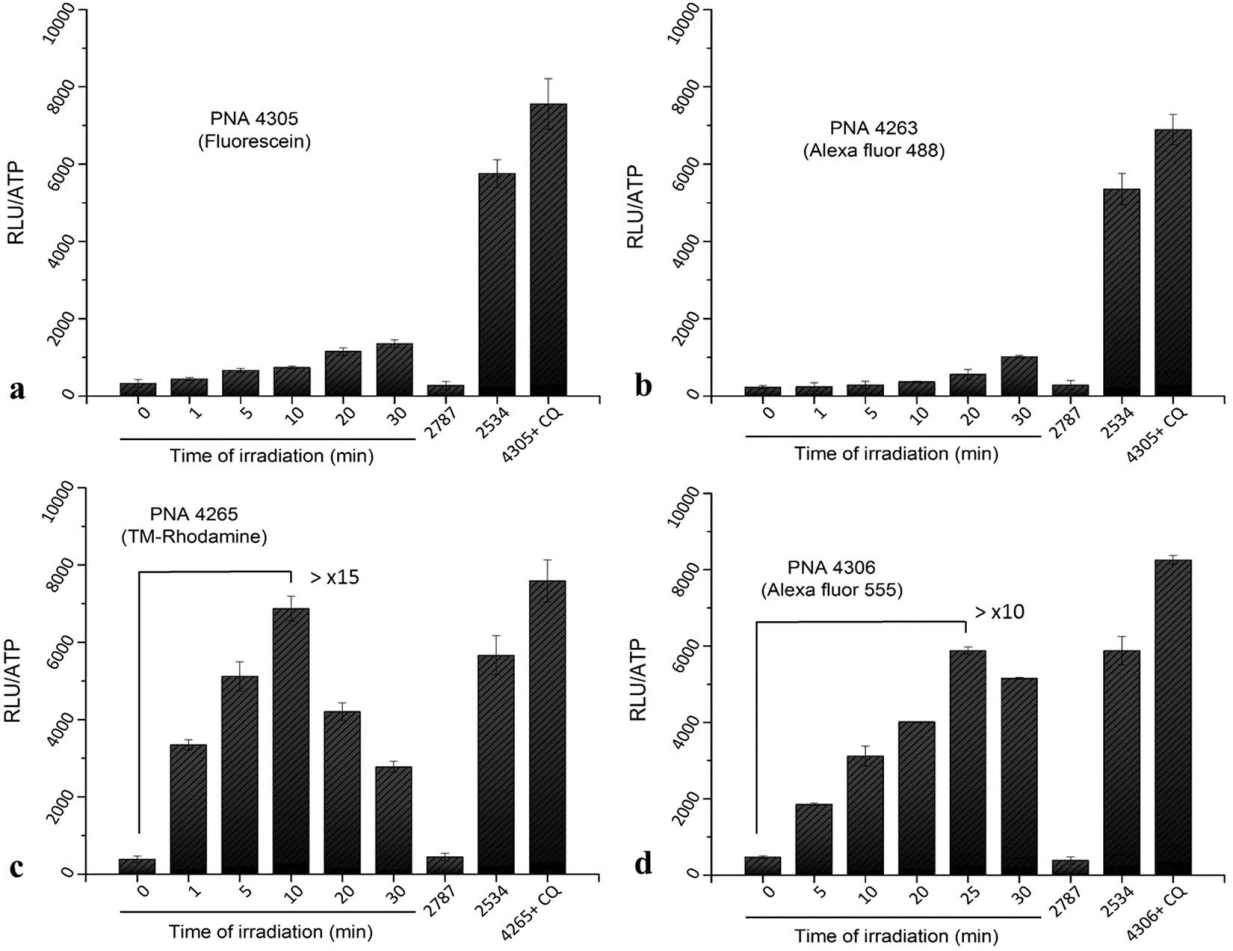
Cellular antisense activity in HeLa pLuc705 cells of PNAs 4305, 4263, 4265, 4306, 2787 and 2534. After transfection of the cells with 3 µM PNA, irradiation of cells was carried out with the excitation wavelength specific for each fluorophore for 1, 5, 10, 20, 25 and 30 min. Cells were incubated further for 24 h after irradiation. All the samples were subjected to the luciferase analysis. Luciferase activities were analysed by Bright-Glo reagent (Promega) and are expressed as relative light units (RLU/well) normalized by the ATP. Each data set represents the mean ± SD of triplicate experiment. CQ: chloroquine. TM-Rhodamine: Tetramethylrhodamine.

**Figure 2:**
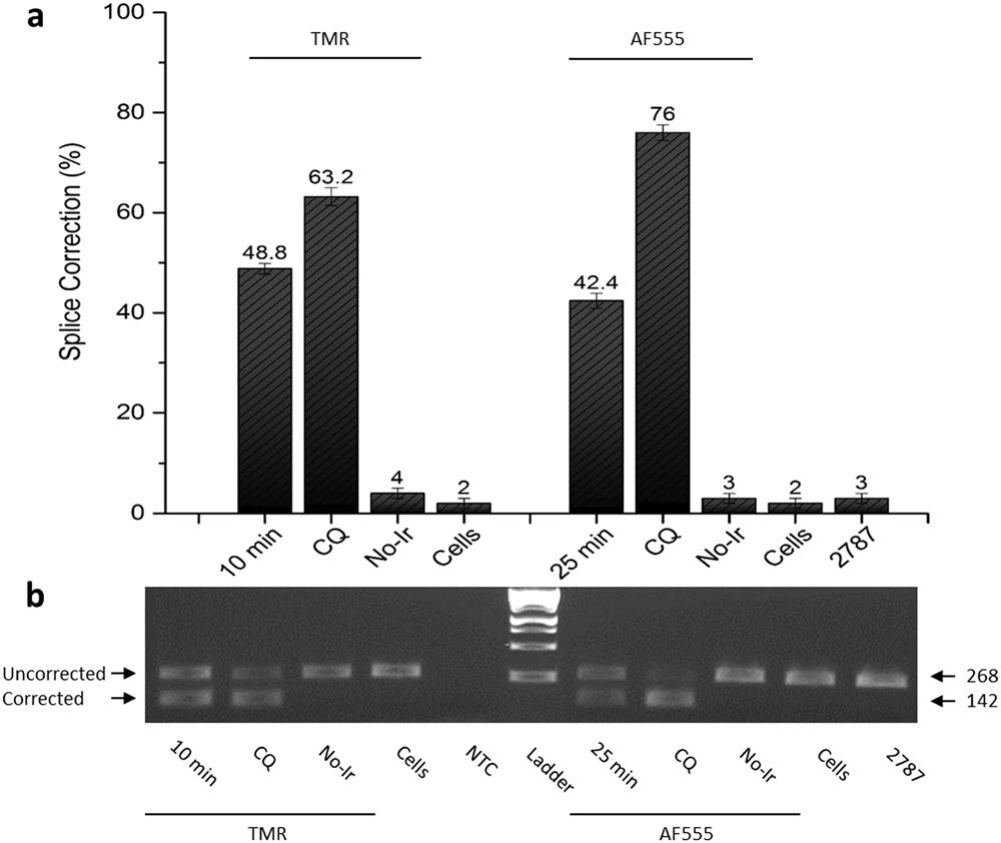
RT-PCR analysis of the splicing correction of pre-mRNAs for PNAs 4265 and 4306. Total RNA was extracted from the cells after the PNA treatment and subjected to RT-PCR analysis. (**a**) Numbers on top of each column indicate the amount of the corrected form relative to the sum of corrected form and uncorrected form. (**b**) Electrophoresis gel image with arrows indicating the 268 bp fragment without splicing correction for the uncorrected gene, and the 142 bp correctly spliced fragment for corrected mRNA. No-Ir: No Irradiation. NTC: no template control.

The enhancing effect of radiation on the antisense luciferase activation was determined in an irradiation dose dependence experiment, and compared to the effect of the endosomolytic agent CQ (Fig. 1). The results clearly demonstrate that the antisense activity of the octaarginine-TMR-PNA (PNA4265) was increased more than 15-fold at an irradiation optimum of 10 min (Fig. 1c). For octaarginine-AF555-PNA (PNA4306), more than 10-fold increase in the antisense activity was observed at 25 min irradiation (Fig. 1d). Most interestingly, the activity for TMR conjugated PNA reached nearly the same level as obtained by the endosomolytic CQ enhancement at 100 μM. Furthermore, the activity was fully comparable to that of the highly active TAT-Lys(Deca)-PNA conjugate (PNA2534)^31^. In contrast, the irradiation dependent enhancement of antisense activity in case of the corresponding fluorescein and AF488 conjugated PNAs was very limited (Fig. 1a and b), although the effect without irradiation in the presence of CQ was similar to that of the TMR and AF555 conjugates. These results were further corroborated by the RT-PCR based, direct determination of mRNA splice correction by TMR and AF555 conjugates (Fig. 2).

### Cytotoxic effects

**Figure 3:**
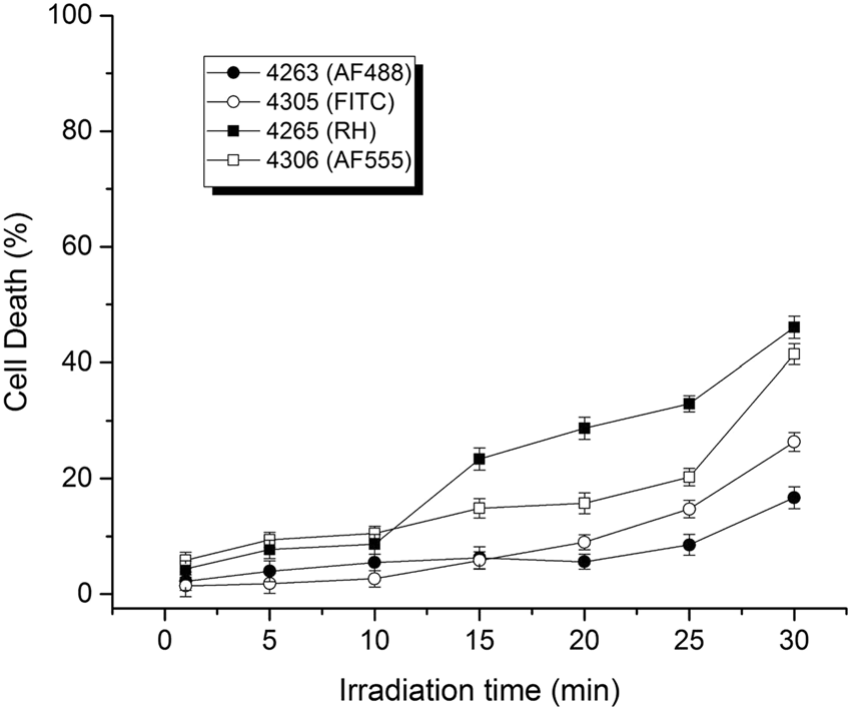
Effect of irradiation time on cellular toxicity of PNA conjugates. Cellular toxicity was analysed by the CytoTox-ONE™ assay (Promega) and the data were normalized to the average value of non-PNA treated sample set as 0% cell death and lysed cells with passive lysis buffer consider as 100% cell death.

The observation of an optimum light dose observed for TMR (PNA 4265) and AF555 (PNA 4306) conjugated PNAs (Fig. 1c and d) most likely reflects a phototoxic effect, and therefore a light dose toxicity study was performed (Fig. 3). These results clearly indicate that all four conjugates exhibit phototoxicity, and this toxicity was higher for the TMR and AF555 conjugates, reaching almost 50% at 30 min irradiation. Thus the decrease in the activity enhancement observed at longer irradiation times is ascribed to phototoxicity.

### Endosomal escape

**Figure 4:**
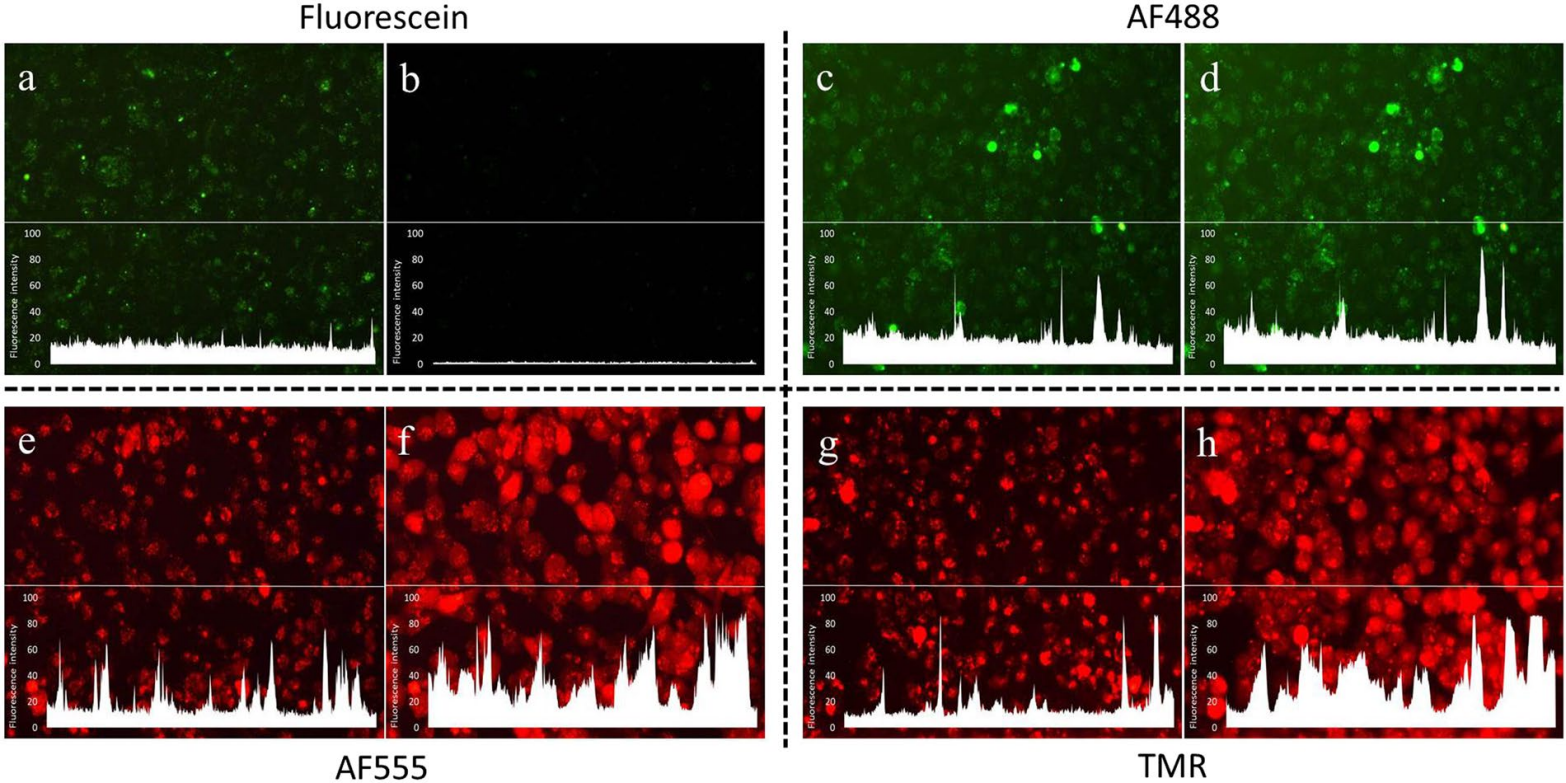
Relocalization of PNA conjugates from endosomes to cytosol upon irradiation. Cells were incubated with the PNA conjugates for 4 h. Cells were imaged before and after the light exposure. Fluorescein conjugate: (**a**) 0 min (**b**) 10 min irradiation. AF488 conjugate: **(c**) 0 min (**d**) 30 min. AF555 conjugate: (**e**) 0 min (**f**) 25 min. TMR conjugate: (**g**) 0 min (h) 10 min. The white graphs on the images represent the fluorescence intensity of each image across the white line drawn at the middle of the image (For easier comparison of before and after irradiation).

**Figure 5:**
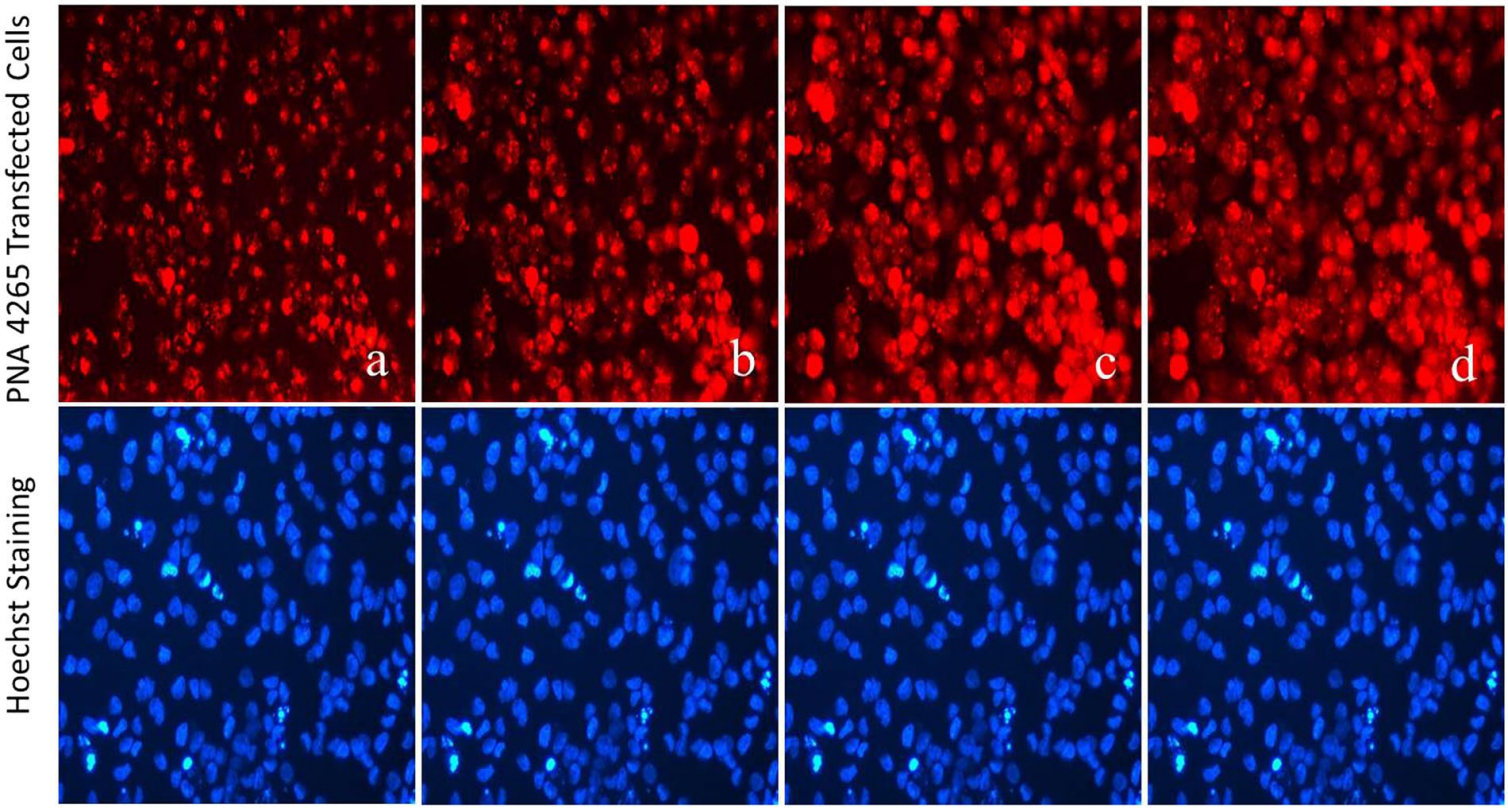
Cellular distribution and relocalization of TMR conjugated PNA. Cells were transfected with 3 µM PNA conjugate. Irradiation was performed at 555 nm (7.87 W/m2) using the N2 filter cube of the fluorescence microscope (Diaplan, leitz). Irradiation time: (**a**) 0 min, (**b**) 3 min, (**c**) 5 min and (**d**) 10 min. Cell nuclei were stained with Hoechst for 15 min. Irradiation and imaging was done using a 20× objective lens. Total cell fluorescence intensity was also measured for each time point (Supplementary Figure S1).

In order to elucidate whether the biological activity results are compatible with a photo-induced endosomal escape mechanism, fluorescence microscopy was used to investigate any changes in the cellular localization of the PNAs upon irradiation. The results clearly show photo-induced relocalization of TMR and AF555 conjugates (Fig. 4e–h) from the endosomes into general cytoplasm, while, no change in the localization of AF488 conjugate is apparent (Fig. 4c and d). In case of the fluorescein conjugate, the fluorescence disappears upon irradiation (Fig. 4a and b). This pattern is entirely compatible with the biological activity. It also provides an explanation for the low PCI activity of the fluorescein conjugate due to the known propensity of this fluorophore for photo-bleaching causing degradation of the chromophore^32^. The low PCI activity seen for the AF488 is likely ascribed to inefficient lipid bilayer disruption in the endosome, and thus inefficient release. For the TMR and AF555 conjugates, an increase in the fluorescence is observed upon irradiation (Figs 4 and 5). This phenomenon is most likely caused by higher self-quenching in the endosomes, where the fluorophore concentration is much higher than in the cytoplasm after release^33^.

**Figure 6:**
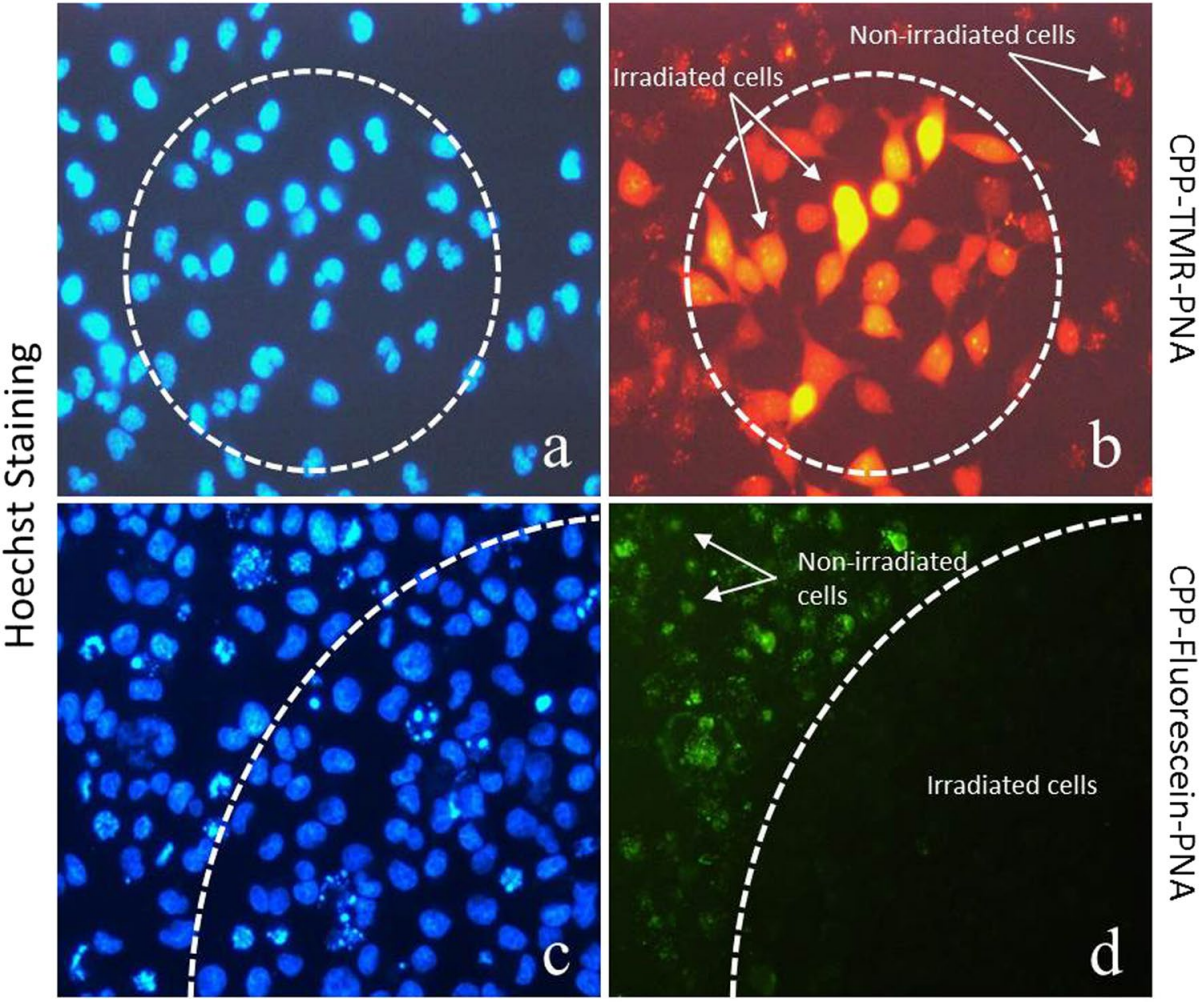
Controlled site specific effect of PCI. Cells were transfected with 3 µM PNA. (**a**,**c**) Cell nuclei were stained with Hoechst for 15 min.; (**b**) Cells transfected with TMR conjugated PNA irradiated at 555 nm; (**d**) Cells transfected with fluorescein conjugated PNA irradiated at 490 nm. Irradiation was confined to the dotted areas (dotted lines). Fluorescence microscope (Diaplan, leitz) with 20× objective lens was used for irradiation and imaging.

Finally, spatial control of the PCI effect was demonstrated by only irradiating a limited area of the cell culture (Fig. 6). Unsurprisingly, the results show irradiation area confined intracellular re-localization of the TMR conjugated PNA, and similarly confined bleaching of the fluorescein conjugated PNA.

### Subcellular distribution of PNA conjugates

**Figure 7:**
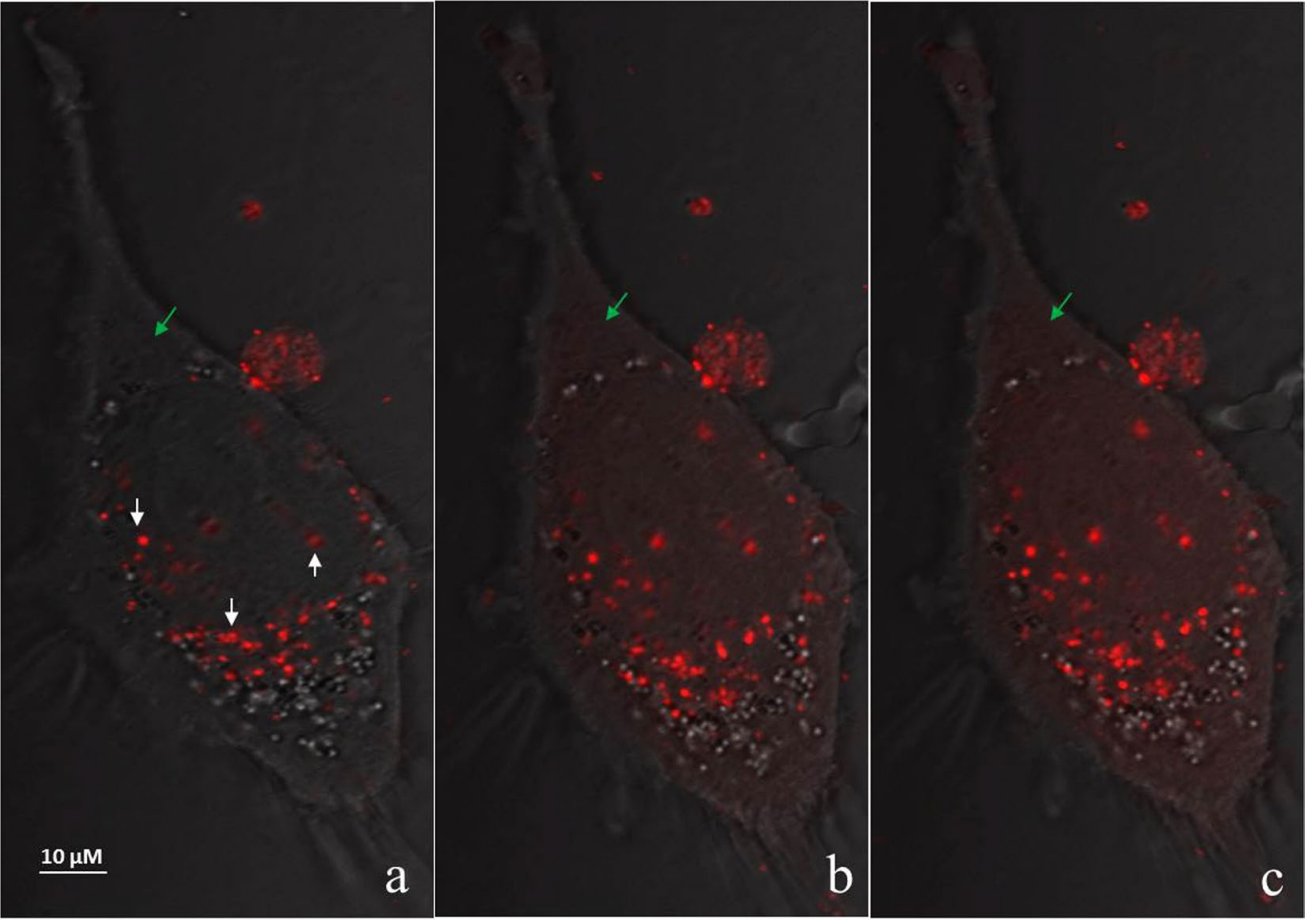
Subcellular distribution study of TMR conjugated PNA using confocal fluorescence microscopy in HeLa pLuc705 cells. Cells were incubated with 3 μM of PNA conjugates, irradiated and imaged using LSM780 with 0.7 mW laser intensity. (**a**) 0 min (**b**) 3 min (**c**) 5 min irradiation. The white arrows indicate endosomes before irradiation. The green arrow shows the fluorescence change of an area before and after irradiation.

In order to study the endosomal escape and the subsequent intracellular distribution of the PNA conjugates in more detail, a confocal microscopy experiment using the TMR conjugated PNA was performed (Fig. 7). These results show a punctate intracellular distribution prior to the irradiation, and in an irradiation dose-dependent manner increase in fluorescence intensity is migrating to the cytoplasm, thus fully compatible with the proposed photo-induced release of endosomally entrapped conjugate.

### ROS/Singlet oxygen formation

**Figure 8:**
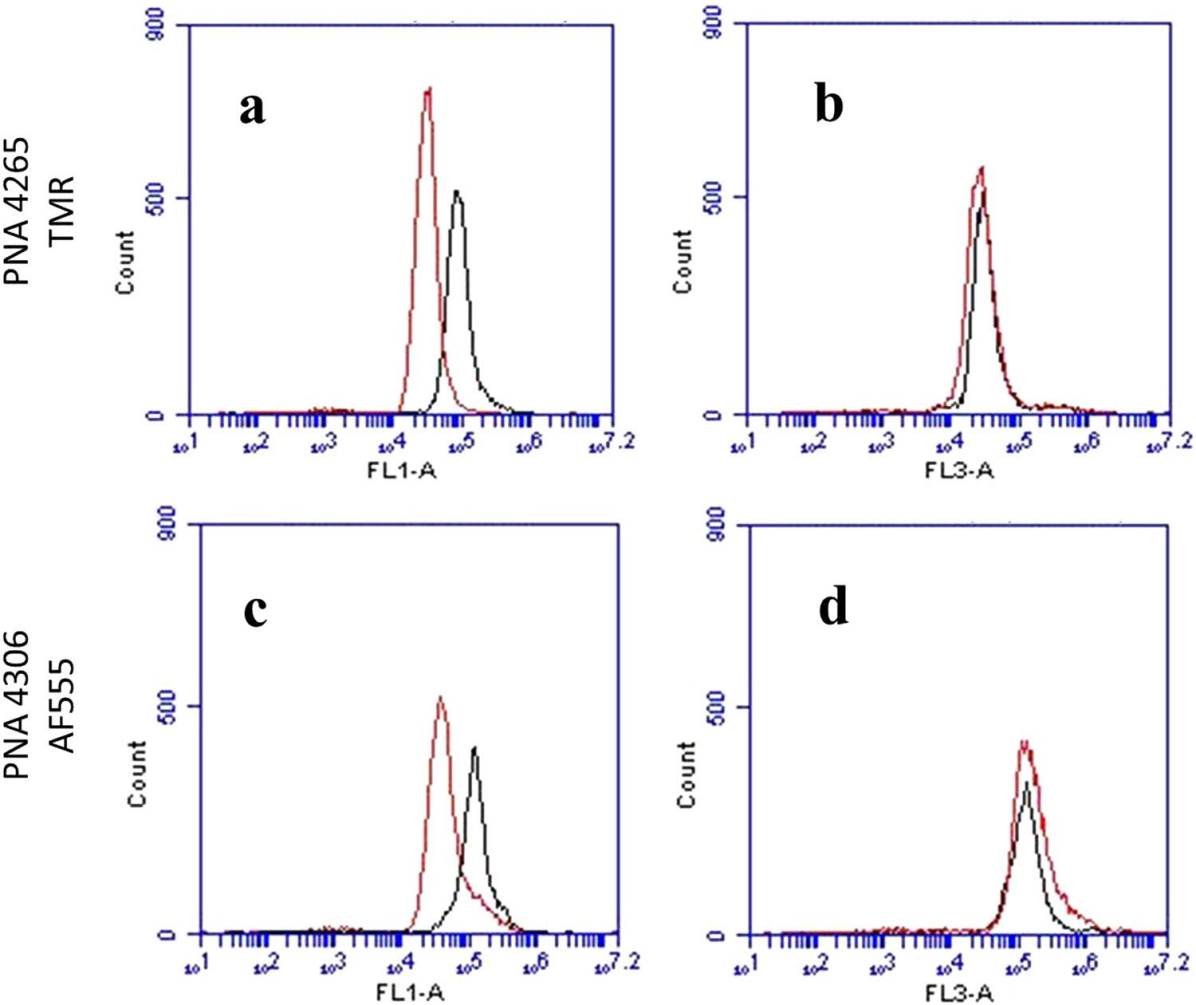
Flow cytometry analysis of ROS/singlet oxygen production. After co-transfection of the cells with PNA and APF, the cells were irradiated. The level of ROS/singlet oxygen was monitored by the APF fluorescence signal in the green channel (FLA-1). A significant increase in singlet oxygen production is clearly visible by change in the peak position toward the higher fluorescent intensities. The mean FLA-1 increased 3-fold for PNA4265 (**a**) and 2.3-fold for PNA4306 (**c**). FLA-3 is the red channel PNA 4265 (**c**) and PNA 4306 (**d**) fluorescence. The red graphs represent before and the black graphs after irradiation.

In order to substantiate the role of singlet oxygen in the endosomal escape process we measured singlet oxygen formation by flow cytometry. Unfortunately, the singlet oxygen sensor green (SOSG) dye does not enter live cells, therefore we resorted to use the APF fluorescent sensor^15,34^, which fluoresce in the green, and thus can be used for studying the red fluorophores. The results presented in Fig. 8 demonstrate that an irradiation dependent green fluorescence appears in the cells. In contrast to SOSG, the APF sensor is not specific for singlet oxygen, and also responds to other reactive oxygen species, in particular hydroxyl radicals^15^. However, in a control experiment (Supplementary Fig. S3a) the APF sensor response was practically unaffected by the presence of the hydroxyl radical quencher dimethyl sulfoxide (DMSO)^15^, and was significantly reduced by the singlet oxygen quencher sodium azide (NaN_3_)^15,35^ (Supplementary Fig. S3b). We therefore ascribe the fluorescence signal to singlet oxygen production from the TMR and AF555 photosensitizer dyes. Using the total cellular fluorescence as a measure of the amount of singlet oxygen formed, the flow cytometry data show that under similar conditions singlet oxygen production is virtually identical for the TMR and AF555 conjugates. However, taking the difference in extinction coefficient at the irradiation wavelength between the two fluorophores (155000 and 92000 cm^−1^ M^−1^ for AF555 and TMR, respectively) into account, the quantum yield for singlet oxygen formation is 1.6-fold higher for TMR compared to AF555. The quantum yield for rhodamine in aqueous solution is reported to be 0.02^36^, and thus from our data we estimate a quantum yield for AF555 for singlet oxygen production of approximately 0.01. The relative quantum yield quantification in the presence of the singlet oxygen sensor green probe also supports these conclusions (Supplementary Table S1).

**Figure 9:**
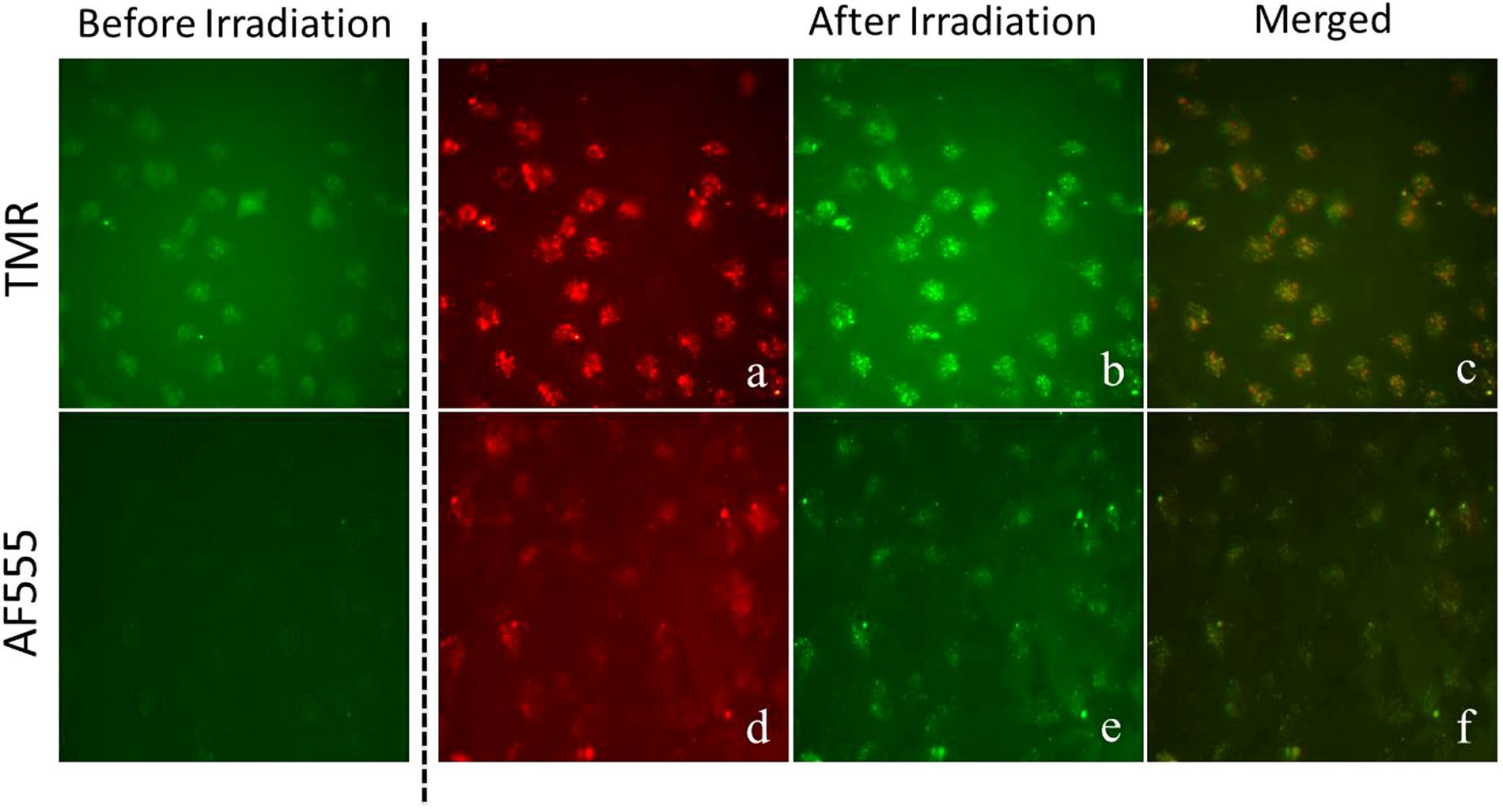
Fluorescence microscope images of unfixed HeLa pLuc705 cells following treatment with 3 µM TMR (**a**–**c**) and AF555 (**d**–**f**) conjugated PNAs and 10 µM APF. After 4 h incubation, the cells were washed twice with phosphate buffered saline (PBS), fresh medium was added and the cells were irradiated at 555 nm. Images were taken at 40× magnification using a Diaplan, Leitz fluorescence microscope.

Fluorescence microscopy analysis (Fig. 9) showed that APF green fluorescence is concentrated in areas (presumably endosomes) where the PNA is also present, as indicated by high degree of co-localization of green and red (i.e. yellow) fluorescence. Therefore, these experiments directly support the singlet oxygen mediated endosomal escape mechanism.

**Figure 10:**
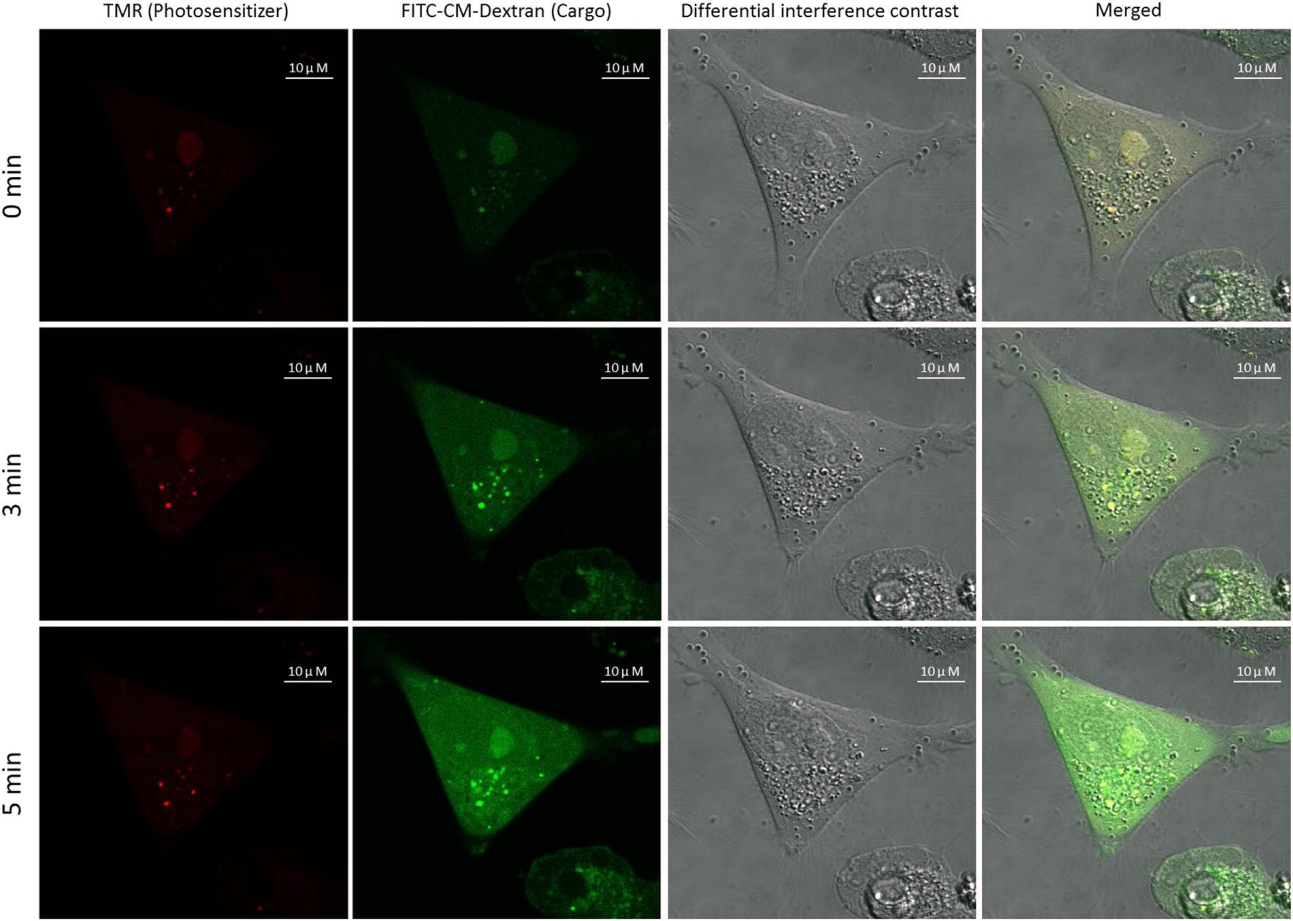
Endosomal release of FITC-CM-Dextran by TMR conjugate PNA photodynamic activity. 3 µM TMR conjugate PNA and 12 µM FITC-CM-Dextran were used as a mixture for transfection of the cells. Irradiation at 555 nm for 0, 3 or 5 min was performed using LSM780 fluorescence microscopy with 0.7 mW laser intensity.

As a final demonstration of endosomal disruption by the CPP-fluorophore-PNA conjugate, we used fluorescein isothiocyanate-carboxymethyl-dextran (FITC-CM-Dextran) as fluid phase marker for endosomal uptake and asked whether this PNA conjugate independent marker would also be released upon irradiation of CPP-fluorophore-PNA conjugate treated cells. The results presented in Fig. 10 show that this was indeed the case, thus indicating the occurrence of general endosome leakage.

It is worth noting that only a smaller fraction of the FITC-CM-Dextran was released from the endosomes in this experiment. This limited release of FITC-CM-Dextran resembles that observed for the TMR conjugated PNA (Fig. 7), and indicates that the process is inherently inefficient and/or that more extensive damage to the endosomes is detrimental (toxic) to the cell, as was observed for longer irradiation times. Taken together, the present results show that octaarginine-PNA conjugated to TMR (and to a lesser extent AF555) fluorophore are effectively, but quantitatively limited, escaping endosomes upon long wavelength irradiation, thus yielding cellular antisense effects comparable to those obtained by the optimal CQ assisted release, but much less than seen for the lipofection delivery^37^.

The poor effect of the fluorescein conjugate is due to the rapid photobleaching (degradation of the fluorophore), while it is interesting that the corresponding AF488 conjugate exhibited comparably very poor photo-assisted activity as well as endosomal escape. This could be due to a low quantum yield for singlet oxygen formation, which unfortunately is not available. However, a relative comparison of singlet oxygen production between fluorescein and AF488 labelled PNAs clearly indicate that these two compounds have comparable quantum yields (0.01–0.03 versus 0.015–0.04, respectively) (Supplementary Table S1), which therefore cannot explain the large difference in cellular activity. Interestingly, we also note that the lipophilicity of the TMR fluorophore is much higher (log P (octanol/water) = 2.92^38^, than that of the AF488 (log P = −1.44^38^). Since the half-life of singlet oxygen is very short (∼0.01–0.04 μs), thereby limiting the action radius to 10–20 nm^16^ compared to the 500 nm size of the endosome, the localization of the fluorophore in the endosome may significantly affect the efficiency of the membrane damage. Therefore, the three orders of magnitude lower lipophilicity of the AF488 fluorophore compared to the rhodamine would result in similarly decreased membrane localization (insertion) of the fluorophore and thus probability of membrane lipid oxidation by the photo-generated singlet oxygen. Hence, we conclude that the low PCI efficiency of the AF488 conjugated compound is predominantly due to the low lipophilicity and thus membrane association of the AF488 chromophore.

## Conclusion

In conclusion, our results show a correlation between endosomal escape and antisense activity and in parallel a correlation to the localized singlet oxygen formation. The results further demonstrate that TMR (and to a lesser extent AF555) conjugated octaarginine PNA is as effectively delivered to the cytosol compartment by PCI as by CQ assisted delivery, and indicate that efficient photodynamic endosomal escape is strongly dependent on photochemical quantum yield for singlet oxygen formation, photostability as well as lipophilicity of the chromophore.

In relation to possible future photodynamic antisense therapy modalities, the present study is relevant for the general mode of action at the cellular level and for optimizing the characteristics of the photosensitizers. For therapeutic applications, near infrared dyes should provide some clear advantages due the deeper tissue penetration of the activating light. It is also worth mentioning, that although significant (PNA) antisense activity enhancement (>10-fold) can be achieved by PCI delivery as shown here as well as in the previous studies^5^, the efficacy in cell culture in terms of antisense agent dose is still more than two orders of magnitude lower than that achievable using lipofection technology^37^.

## Methods

### Synthesis of PNAs

PNA synthesis was carried out as reported previously^39^. Cationic peptides were linked to PNA at the N-terminal through a cysteine via continuous solid phase synthesis. Fluorescent dyes as maleimide derivatives were coupled to the cysteine. The PNA conjugates were purified by HPLC and characterized by MALDI-TOF mass spectrometry. The PNAs were lyophilized and stored at 4 °C until use. The sequences of the PNAs are listed in Table 1.

**Table 1:** PNA conjugates list.

#	PNA Sequence (N - > C)	Mass found	Mass calculated	Purity (%)
2534	^a^TAT-Lys(^b^Deca)-Gly- CCT CTT ACC TCA GTT ACA-NH_2_	6809	6807.9	98
2787	H- (D-Arg)_8_-Gly- CCT CTT ACC TCA GTT ACA-NH_2_	6080	6072.0	96
4263	H-(D-Arg)_8_-Cys(^c^AF488)-CCT CTT ACC TCA GTT ACA-NH_2_	6819	6837.7	98
4305	H-(D-Arg)_8_-Cys(^d^Flm)-CCT CTT ACC TCA GTT ACA-NH_2_	6551	6544.5	98
4265	H-(D-Arg)_8_-Cys(^e^TMR)-CCT CTT ACC TCA GTT ACA-NH_2_	6615	6600.1	97
4306	H-(D-Arg)_8_-Cys(^f^AF555)-CCT CTT ACC TCA GTT ACA-NH_2_	7074	7368*	98

^a^TAT: Trans-activator of Transcription (GRKKRRQRRRPPQ), ^b^Deca: Decanoic acid, ^c^AF488: Alexa Fluor 488, ^d^Flm: Fluorescein, ^e^TMR: Tetramethylrhodamine, ^f^AF555: Alexa Fluor 555, ^*^The exact mass of AF555 is not revealed by the company.

### Cell culture

HeLa pLuc705 cells were purchased from Gene Tools (USA). Cells were grown in RPMI-1640 medium (Sigma) supplemented with 10% fetal bovine serum (FBS), 1% glutamax (Gibco), penicillin (100 U/ml) and streptomycin (100 µg/ml) at 37 °C in humidified air with 5% CO_2_. For the following studies cells were seeded in four format culture vessels (based on the needs of the experiments) 16 h before treatment. The cells were seeded at 2 × 10^4^ cells/well 96-well plates. For the studies in 4-well chamber slid system and 24-well plate format, 1.2 × 10^5^ cells/well were seeded. For the confocal study the cells were seeded at 2 × 10^5^ cell/ml in petri dishes (TC, Dish 60 × 15, VENTS NUNCLON) incorporating a coverslip window (42 mm, Menzel-Glaser, Thermo Scientific) on the underside designed for inverted microscopy (LSM 780 confocal microscope, Zeiss).

### PNA treatment

Cells were treated with 3 µM of PNA (final concentration) in OPTI-MEM (Gibco) 16 h after seeding: 100 µL/well for 96-well plate, 500 µL/well for 4-well chamber slid, 500 µL/well for 24-well plate and 1000 µL/well for petri dish was used. Cells were incubated with PNA for 4 h. Then the medium was replaced with the same volume of fresh growth medium (RPMI-1640 containing 10% FBS and 1% glutamax) and incubated for 2 h.

### PCI treatment and microscopy study

#### Microscope irradiation

Cells were seeded in 4-well chamber slid system and treated with PNA as described above. The cells were subsequently irradiated with blue or green light generated from the fluorescence microscope (20× objective lens) (Diaplan, leitz). Emission and excitation wavelengths of the blue and green lights are given in the Supplementary Table S2 (a). Blue light of the microscope with 33.58 W/m^2^ intensity was used for the green fluorophores excitation and generated by H3 filter cube. Also green light with 7.87 W/m^2^ intensity was generated by N2 filter cube and has been used for the red fluorophores excitation (Supplementary Table S2 (b)). Images were collected using an infinity 2-2 (Lumenera) CCD camera. Images were acquired using the standard filter sets: H3: Violet/blue (Beam splitting mirror: 520) and N2: Green (Beam splitting mirror: 580). The light intensity of the fluorescence microscope was measured using the EPP2000 portable spectrometers (StellarNet, Inc.) in 1 mm distance from the lens for the microscope, where the samples are mounted.

### Light emitting diode (LED) projector Irradiation

Cells were cultured in a 4-well chamber system, were treated with PNA and subsequently irradiated with blue or green light generated from an LED Projector lamp (Supplementary Fig. S2) and imaged immediately after irradiation. The light intensity of LED projector lamp was measured at 6 mm distance from the lens, where the samples are mounted. The light intensity of the blue and green light was 8.68 and 3.95 W/m^2^ respectively (Supplementary Table S2 (c)).

### Confocal fluorescence laser scanning microscopy

Cells were cultured in petri dish format and treated with TMR conjugated PNA. Prior to the confocal microscopy (LSM 780 confocal microscope, Zeiss) cells were washed three times with PBS (37 °C). For live confocal fluorescence microscopy the cells were incubated with phenol red-free RPMI-1640 (Thermo Fisher Scientific). Fluorescence images were taken during the irradiation process. The light treatment was carried out on the stage by using 43HE filter cubes for mercury lamp (HXP120) with 555 nm laser excitation and 0.7 mW laser power.

### Luciferase activity assay

Cells were seeded in 96 well plates, incubated with PNA and then subjected to irradiation (0–30 min) using the LED projector lamp light source. At day three (24 h after irradiation) cells were lysed with passive lysis buffer (Promega) and subjected to the luciferase activity analysis by using the Bright-Glo luciferase assay system (Promega) based on the manufacturer’s protocol. Luminescent readings obtained by the Bright-Glo luciferase assay system were background-subtracted and normalized for the cell viability by the CellTiter-Glo ATP measurement system (Promega) based on the manufacturer protocol.

### Cytotoxicity

Supernatants from the 96-well plates of the luciferase activity assay (previous section) were collected and subjected to cytotoxicity test. The supernatant was transferred to a 96-well plate and was subjected to a lactate dehydrogenase (LDH) assay using CytoTox-ONE (Promega). The procedure was performed according to the manufacturer’s instructions. The measured absorbance is presented as relative cellular toxicity. In this experiment absorbance from cells without PNA treatment was set as 0% cell death and lysed cells with passive lysis buffer consider as 100% cell death.

### RT-PCR

The RT-PCR was performed as previously published^22^. Briefly total RNA was extracted from the cellular lysate prepared for the luciferase assay by using RNeasy Mini kit (Qiagen) and subjected to RT-PCR analysis. RT-PCR was performed using OneStep RT-PCR kit (Qiagen). A total of 2 ng RNA was used for each 20 µL RT-PCR reaction. Primers used in RT-PCR were as follows: forward primer: 5′-TTGATATGTGGATTTCGAGTCGTC-3′; reverse primer: 5′-TGTCAATCAGAGTGCTTTTGGCG-3′. The RT-PCR program was as follows: (55 °C, 35 min) × 1 cycle, (95 °C, 15 min) × 1 cycle, (94 °C, 0.5 min; 55 °C, 0.5 min; 72 °C, 0.5 min) × 29 cycles. RT-PCR products were analysed on 2% agarose gel with 1 × Tris/Borate/EDTA (TBE) buffer and visualized by ethidium bromide staining. Gel image were captured by Gene Flash gel documentation (Syngene) and analysed by UN-SCAN-IT software (Silk Scientific Corporation)^40^.

### Singlet oxygen determination

#### Flow cytometry

ROS/Singlet oxygen production by the red fluorophores (TMR and AF555) upon irradiation was measured using the green fluorescence ROS/singlet oxygen probe aminophenyl fluorescein (APF, 2-[6-(4′-Amino)phenoxy-3H-xanthen-3-on-9-yl] benzoic acid, C26H17NO5, Life Technologies). Cells were cultured and treated with PNA in the 24-well plate format. After 4 h, the PNA solution was removed and 500 µL/well of pre-heated growth medium was added and incubation continued for 1 h. Then, all medium was removed by aspiration, and 500 µL/well of pre-heated OPTI-MEM containing 0.5 µL APF (10 µM final concentration) was added (according to the manufacturer’s recommended dose). Plates were incubated 1 h at 37 °C, then the OPTI-MEM removed and the cells were washed with pre-warmed growth medium. Growth medium was added and cells were irradiated with the LED projector light source for 10 and 25 min for TMR and AF555 conjugated PNAs, respectively. Then, cells were harvested with Trypsin-EDTA and immediately subjected to flow cytometric analysis using BD Accuri™ Flow Cytometer to determine the mean APF fluorescence intensity after gating in irradiated and non-irradiated cells.

### Co-localization analysis

Cells were cultured in 24-well plates and were treated with 3 µM of TMR and AF555 conjugated PNA for 4 h, the medium was removed, new medium was added, and the cells were left in the incubator for 1 h. Then, the medium was replaced by pre-warmed medium containing 10 µM APF and incubated at 37 °C for 1 h. Cells were gently washed in pre-warmed growth medium, then fresh medium was added, and the plate was placed on the fluorescence microscope and irradiated by N2: Green (Beam splitting mirror: 580) with 3.95 W/m^2^.

### Data availability

All data generated or analysed during this study are included in this published article (and its Supplementary Information files).

## Electronic supplementary material

Supplementary Information

## References

[1] Turner JJ (2005). Cell-penetrating peptide conjugates of peptide nucleic acids (PNA) as inhibitors of HIV-1 Tat-dependent trans-activation in cells. Nucleic Acids Res.

[2] Shiraishi T, Pankratova S, Nielsen PE (2005). Calcium ions effectively enhance the effect of antisense peptide nucleic acids conjugated to cationic tat and oligoarginine peptides. Chem Biol.

[3] Shiraishi T, Nielsen PE (2006). Enhanced delivery of cell-penetrating peptide-peptide nucleic acid conjugates by endosomal disruption. Nature protocols.

[4] Abes S (2006). Endosome trapping limits the efficiency of splicing correction by PNA-oligolysine conjugates. J Control Release.

[5] Shiraishi T, Nielsen PE (2006). Photochemically enhanced cellular delivery of cell penetrating peptide-PNA conjugates. FEBS Lett.

[6] Selbo PK, Sivam G, Fodstad O, Sandvig K, Berg K (2001). *In vivo* documentation of photochemical internalization, a novel approach to site specific cancer therapy. International journal of cancer. Journal international du cancer.

[7] Lu HL, Syu WJ, Nishiyama N, Kataoka K, Lai PS (2011). Dendrimer phthalocyanine-encapsulated polymeric micelle-mediated photochemical internalization extends the efficacy of photodynamic therapy and overcomes drug-resistance *in vivo*. J Control Release.

[8] Oliveira S, Hogset A, Storm G, Schiffelers RM (2008). Delivery of siRNA to the target cell cytoplasm: photochemical internalization facilitates endosomal escape and improves silencing efficiency, *in vitro* and *in vivo*. Current pharmaceutical design.

[9] Theodossiou TA, Goncalves AR, Yannakopoulou K, Skarpen E, Berg K (2015). Photochemical internalization of tamoxifens transported by a “Trojan-horse” nanoconjugate into breast-cancer cell lines. Angew Chem Int Ed Engl.

[10] Gederaas OA (2015). Photochemical internalization of bleomycin and temozolomide–*in vitro* studies on the glioma cell line F98. Photochem Photobiol Sci.

[11] Boe SL, Hovig E (2013). Enhancing nucleic acid delivery by photochemical internalization. Therapeutic delivery.

[12] Ohtsuki T (2015). The molecular mechanism of photochemical internalization of cell penetrating peptide-cargo-photosensitizer conjugates. Sci Rep.

[13] Hwang JY (2012). Photoexcitation of tumor-targeted corroles induces singlet oxygen-mediated augmentation of cytotoxicity. J Control Release.

[14] Muthukrishnan N, Johnson GA, Erazo-Oliveras A, Pellois JP (2013). Synergy between cell-penetrating peptides and singlet oxygen generators leads to efficient photolysis of membranes. Photochemistry and photobiology.

[15] Price M, Reiners JJ, Santiago AM, Kessel D (2009). Monitoring singlet oxygen and hydroxyl radical formation with fluorescent probes during photodynamic therapy. Photochemistry and photobiology.

[16] Moan J, Berg K (1991). The Photodegradation of Porphyrins in Cells Can Be Used to Estimate the Lifetime of Singlet Oxygen. Photochemistry and photobiology.

[17] Mellert K, Lamla M, Scheffzek K, Wittig R, Kaufmann D (2012). Enhancing endosomal escape of transduced proteins by photochemical internalisation. PloS one.

[18] Gillmeister MP, Betenbaugh MJ, Fishman PS (2011). Cellular trafficking and photochemical internalization of cell penetrating peptide linked cargo proteins: a dual fluorescent labeling study. Bioconjugate chemistry.

[19] Jerjes W, Upile T, Radhi H, Hopper C (2011). Photodynamic therapy vs. photochemical internalization: the surgical margin. Head & neck oncology.

[20] Huang Z (2005). A review of progress in clinical photodynamic therapy. Technol Cancer Res Treat.

[21] Folini M (2003). Photochemical internalization of a peptide nucleic acid targeting the catalytic subunit of human telomerase. Cancer research.

[22] Folini M (2007). Photochemically enhanced delivery of a cell-penetrating peptide nucleic acid conjugate targeting human telomerase reverse transcriptase: effects on telomere status and proliferative potential of human prostate cancer cells. Cell proliferation.

[23] Boe S, Hovig E (2006). Photochemically induced gene silencing using PNA-peptide conjugates. Oligonucleotides.

[24] Boe SL, Longva AS, Hovig E (2011). A novel photosensitizer for light-controlled gene silencing. Nucleic acid therapeutics.

[25] Marlin F (2012). Flavin conjugates for delivery of peptide nucleic acids. Chembiochem.

[26] Kang SH, Cho MJ, Kole R (1998). Up-regulation of luciferase gene expression with antisense oligonucleotides: implications and applications in functional assay development. Biochemistry.

[27] Shiraishi, T. & Nielsen, P. E. In *Delivery Technologies for Biopharmaceuticals* 305–338 (John Wiley & Sons, Ltd, 2009).

[28] Mitchell DJ, Kim DT, Steinman L, Fathman CG, Rothbard JB (2000). Polyarginine enters cells more efficiently than other polycationic homopolymers. J Pept Res.

[29] Schmidt N, Mishra A, Lai GH, Wong GC (2010). Arginine-rich cell-penetrating peptides. FEBS Lett.

[30] Rothbard JB (2002). Arginine-rich molecular transporters for drug delivery: role of backbone spacing in cellular uptake. J Med Chem.

[31] Koppelhus U, Shiraishi T, Zachar V, Pankratova S, Nielsen PE (2008). Improved cellular activity of antisense peptide nucleic acids by conjugation to a cationic peptide-lipid (CatLip) domain. Bioconjugate chemistry.

[32] Song L, Hennink EJ, Young IT, Tanke HJ (1995). Photobleaching kinetics of fluorescein in quantitative fluorescence microscopy. Biophys J.

[33] Swiecicki J-M (2016). How to unveil self-quenched fluorophores and subsequently map the subcellular distribution of exogenous peptides. Scientific Reports.

[34] Setsukinai K, Urano Y, Kakinuma K, Majima HJ, Nagano T (2003). Development of novel fluorescence probes that can reliably detect reactive oxygen species and distinguish specific species. The Journal of biological chemistry.

[35] Bancirova M (2011). Sodium azide as a specific quencher of singlet oxygen during chemiluminescent detection by luminol and Cypridina luciferin analogues. Luminescence: the journal of biological and chemical luminescence.

[36] Bunting JR (1992). A Test of the Singlet Oxygen Mechanism of Cationic Dye Photosensitization of Mitochondrial Damage. Photochemistry and photobiology.

[37] Shiraishi T, Hamzavi R, Nielsen PE (2008). Subnanomolar antisense activity of phosphonate-peptide nucleic acid (PNA) conjugates delivered by cationic lipids to HeLa cells. Nucleic Acids Res.

[38] Ogawa M, Kosaka N, Choyke PL, Kobayashi H (2009). H-type dimer formation of fluorophores: a mechanism for activatable, *in vivo* optical molecular imaging. ACS Chem Biol.

[39] Christensen L (1995). Solid-phase synthesis of peptide nucleic acids. Journal of peptide science: an official publication of the European Peptide Society.

[40] Shiraishi T, Nielsen PE (2004). Down-regulation of MDM2 and activation of p53 in human cancer cells by antisense 9-aminoacridine-PNA (peptide nucleic acid) conjugates. Nucleic Acids Res.

