# Supplementary material for: Effective photo-enhancement of cellular activity of fluorophore-octaarginine antisense PNA conjugates correlates with singlet oxygen formation, endosomal escape and chromophore lipophilicity
Source: Sci Rep. 2018 Jan 12;8:638. doi: 10.1038/s41598-017-18947-x (PMC5766568; doi:10.1038/s41598-017-18947-x)
Supplement: Supplementary file 1 — Supplementary Information [file 41598_2017_18947_MOESM1_ESM.pdf]

Effective photo-enhancement of cellular activity of fluorophore-octaarginine antisense PNA conjugates correlates with singlet oxygen formation, endosomal escape and chromophore lipophilicity

Reza Yarani[1], Takehiko Shiraishi[1], Peter E. Nielsen[1*]

[1] Department of Cellular and Molecular Medicine, Faculty of Health and Medical Sciences, University of Copenhagen, Copenhagen, Denmark

Supplementary Information

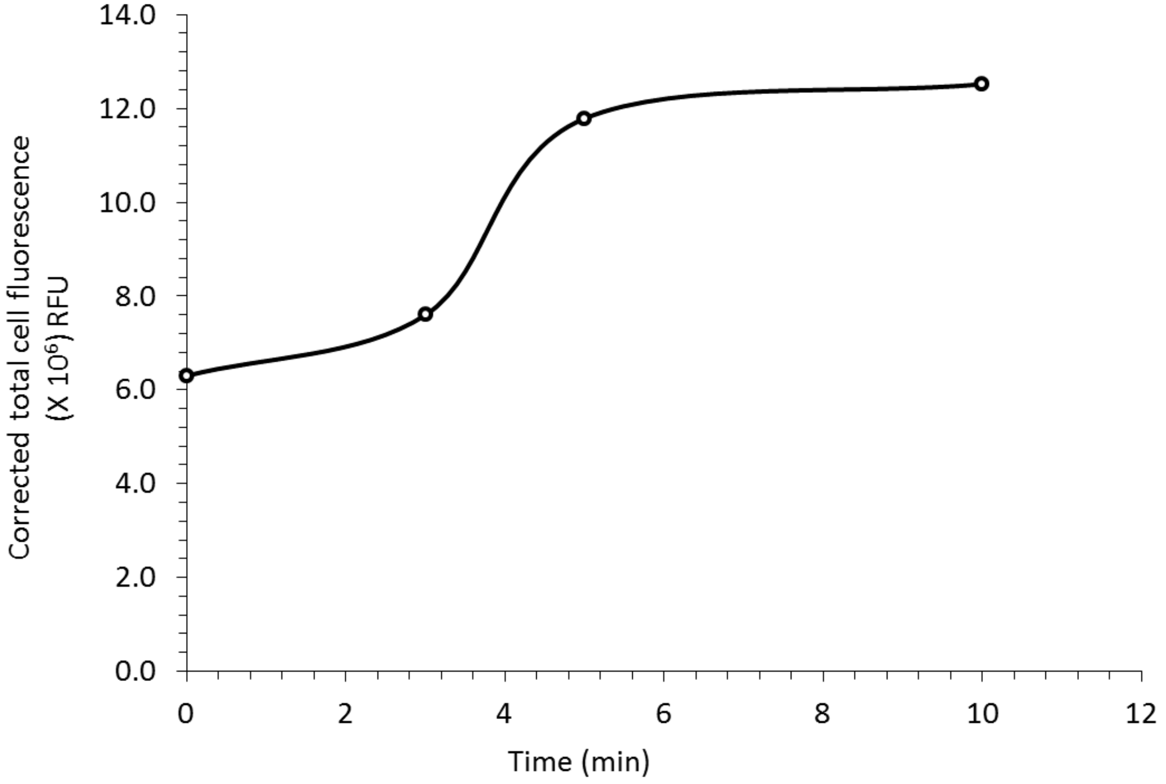

Supplementary Figure S1: Corrected total cell fluorescent intensity of irradiated cells transfected with TMR conjugated PNA. Total fluorescent intensity of the cells was increased through release of fluorescently labeled PNAs from the endosomes upon irradiation. The increase of fluorescent for four time points (0, 3, 5 and 10 min) was quantified using image J software (v1.5g, NIH) after subtracting the background signal by drawing an outline around each image and measure the mean fluorescence for each image. Several adjacent background measurements were recorded, and the average background florescence was calculated for background correction. The total corrected cellular fluorescence (TCCF) was calculated as follows: TCCF = integrated density – (area of selected cell × average of mean fluorescence of background readings).

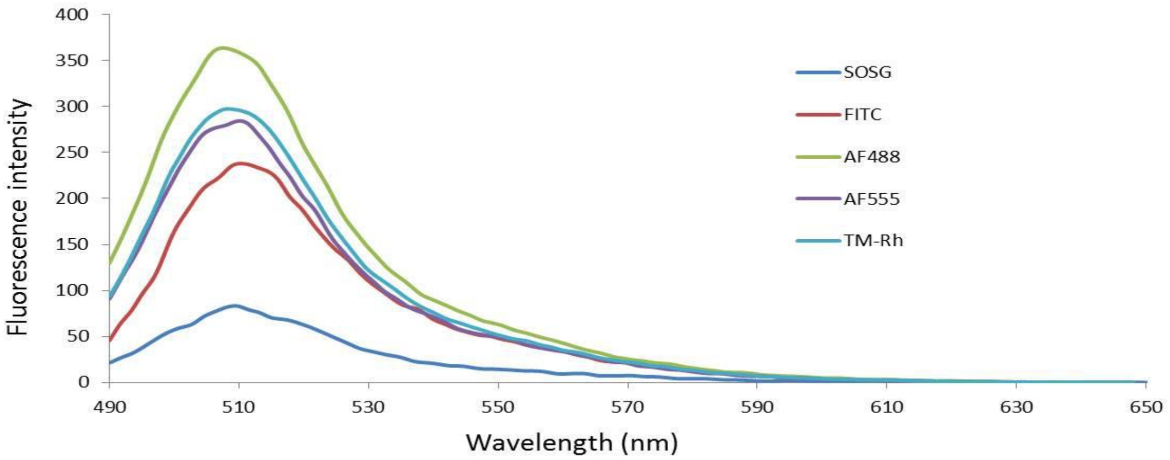

PNA#	$^1O_2$ formation (arbitrary units)	Relative Absorbance at excitation wavelength	$^1O_2$ formation corrected for fluorophore absorbance	Literature $^1O_2$ Quantum yield (ϕ)	Estimated $^1O_2$ Quantum yield (ϕ)
AF488 (4263)	363	0.262	1387	-	0.015<br>0.04
TM-Rh (4265)	297	0.314	947	0.02[1]	0.02<br>0.06
FITC (4305)	232	0.234	992	0.03[2]	0.01<br>0.03
AF555 (4306)	284	1	284	-	0.006<br>0.02

Supplementary Table S1: Relative quantum yield of singlet oxygen production (Φ) measurement for the CPP-fluorophore-PNA conjugates. Φ was calculated in the presence of 1 µM singlet oxygen sensor green (SOSG, Thermo Fisher Scientific), in an aqueous solution containing 0.1 µM PNA conjugates. The fluorescence intensity after irradiation is shown after correcting for the contribution from each of the fluorophores. The measured singlet oxygen formation was normalized to fluorophore absorbance at the irradiation wavelength. The Φ of each conjugate was estimated by using an already known Φ of TMR and FITC [1,2], the normalized singlet oxygen formation, and correcting for the ratio in light intensity between the blue and the green light (factor 2.2). The quantum yields were estimated using published data on rhodamine and fluorescein ref), yielding somewhat different results.

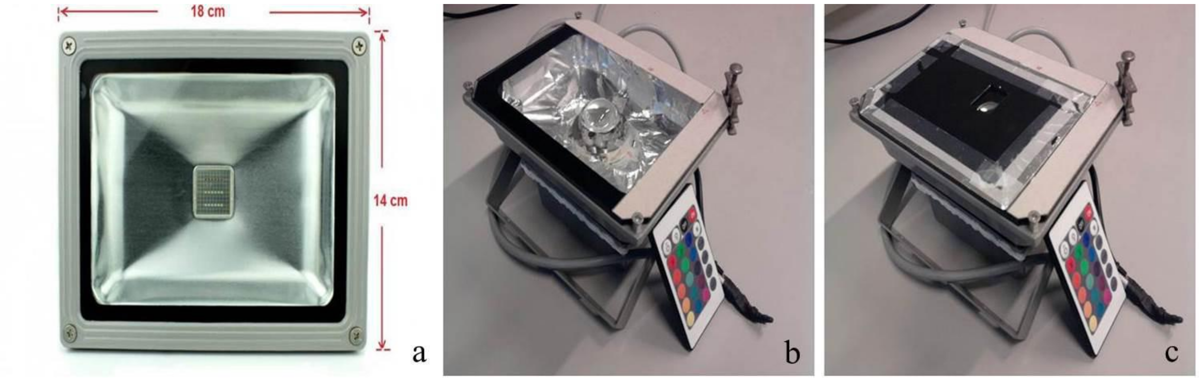

Supplementary Figure S2: Light emitting diode (LED) projector (IP65, 20W, RGB) used as the irradiation source for PCI. (a) Physical dimensions of the LED projector provided by company webpage. (b) A modified version of the LED projector used for the PCI study. (c) Modified projector with light protection cover ready for irradiation of the mounted plates. This modified projector allowed us to irradiate 6 wells of 96-wells plate at the same time.

a

Fluorophores		Excitation (nm)	Emission (nm)
Green	Fluorescein	490	520
	AF 488	495	518
Red	Rhodamine	550	570
	AF 555	555	565

b

Microscope	H3 (Blue)	N2 (Green)
4X magnification	11.83	3.82
20X magnification	33.58	7.87

c

LED projector	Blue	Green
Light intensity	8.68	3.95

Supplementary Table S2: Wavelengths and light intensities table. (a) Excitation and emission wavelength of green and red fluorophores used in this study. Light intensity for both blue and green light (b) microscope and (c) LED projector which are shown as W/m2 unit. Light intensity was measured using EPP2000 portable spectrometers (StellarNet, Inc.) for both instruments on which the samples are mounted.

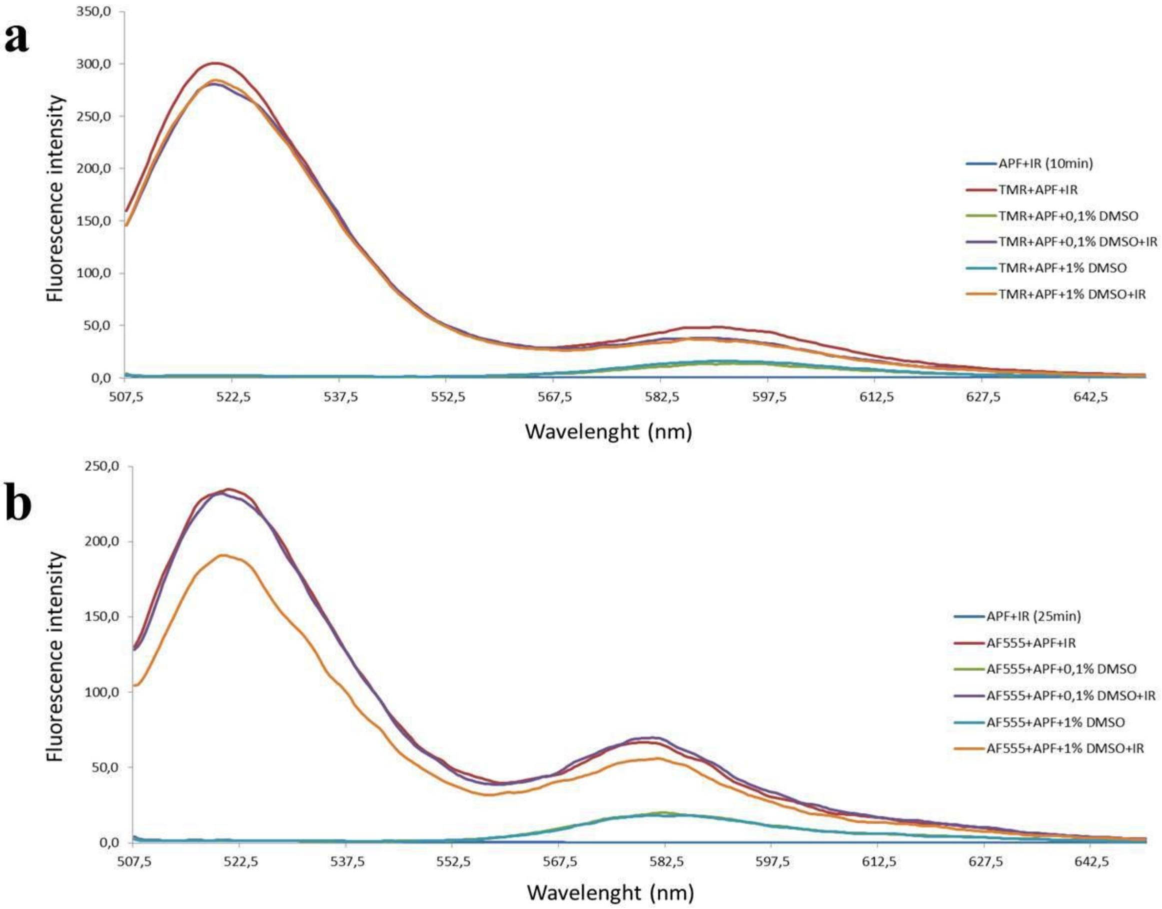

Supplementary Figure S3A: Fluorescence response of APF to the reactive oxygen species produced upon irradiation of TMR (a) and AF555 (b) conjugated PNAs, respectively in the presence of 0.1% and 1% DMSO. Fluorescent intensity of 5 µM APF was measured in a phosphate buffered saline solution containing 1 µM PNA conjugates. After irradiation, a portion of the quenched fluorescence by DMSO represents the contribution of hydroxyl radical, while the remaining signal shows the singlet oxygen derived fluorescence [3,4].

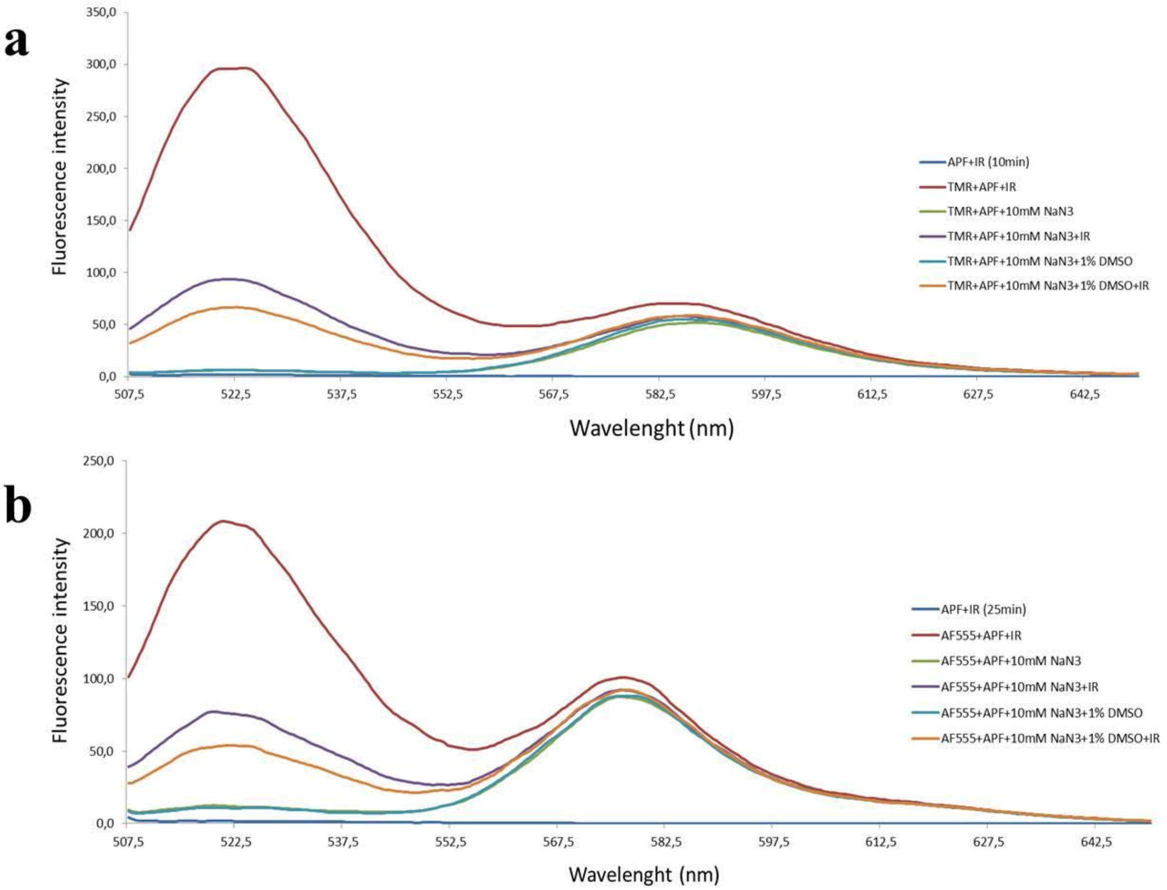

Supplementary Figure S3B: Fluorescence response of APF to the reactive oxygen species produced upon irradiation of TMR (a) and AF555 (b) conjugated PNAs, respectively in the presence 10mM sodium azide (NaN_3) and 1% DMSO. Fluorescent intensity of 5 µM APF was measured in a phosphate buffered saline solution containing 1 µM PNA conjugates. After irradiation, the portion of the quenched fluorescence by NaN_3 represents the contribution of singlet oxygen derived fluorescence [4,5]. Moreover, 1% DMSO was added to quench the signal derived from hydroxyl radical. The results shown in supplementary table S1 and S3 together with the current data confirm that the primary and dominant oxidant generated during this process is singlet oxygen.

Reference:

1. Bunting, J. R. A Test of the Singlet Oxygen Mechanism of Cationic Dye Photosensitization of Mitochondrial Damage. *Photochem Photobiol* **55**, 81-87, doi:DOI 10.1111/j.1751-1097.1992.tb04212.x (1992).
2. Gandin, E., Lion, Y. & Vandevorst, A. Quantum Yield of Singlet Oxygen Production by Xanthene Derivatives. *Photochem Photobiol* **37**, 271-278, doi:DOI 10.1111/j.1751-1097.1983.tb04472.x (1983).
3. Liu, S., Oshita, S., Kawabata, S., Makino, Y. & Yoshimoto, T. Identification of ROS Produced by Nanobubbles and Their Positive and Negative Effects on Vegetable Seed Germination. *Langmuir : the ACS journal of surfaces and colloids* **32**, 11295-11302, doi:10.1021/acs.langmuir.6b01621 (2016).
4. Price, M., Reiners, J. J., Santiago, A. M. & Kessel, D. Monitoring singlet oxygen and hydroxyl radical formation with fluorescent probes during photodynamic therapy. *Photochem Photobiol* **85**, 1177-1181, doi:10.1111/j.1751-1097.2009.00555.x (2009).
5. Bancirova, M. Sodium azide as a specific quencher of singlet oxygen during chemiluminescent detection by luminol and Cypridina luciferin analogues. *Luminescence : the journal of biological and chemical luminescence* **26**, 685-688, doi:10.1002/bio.1296 (2011).

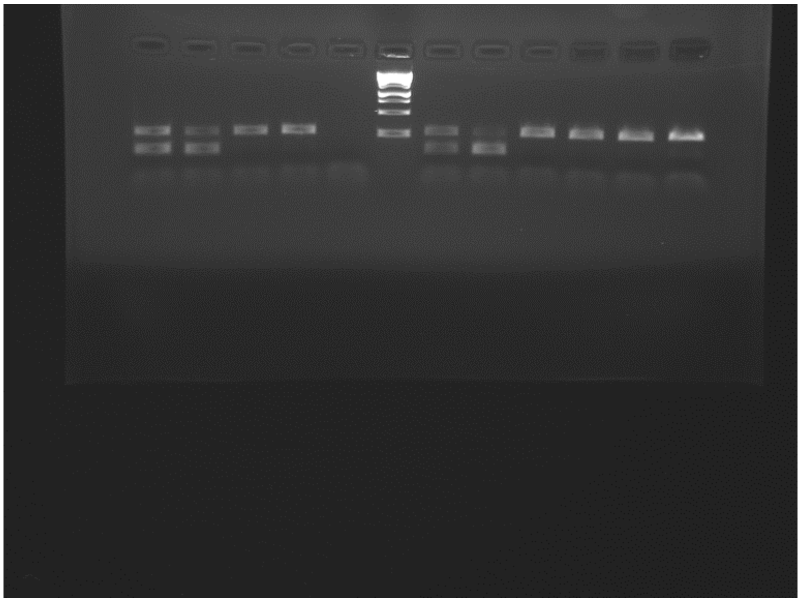

Total, original Image of gel from Figure 2 (b)
